# Sharp force trauma with two katana swords: identifying the murder weapon by comparing tool marks on the skull bone

**DOI:** 10.1007/s00414-020-02372-3

**Published:** 2020-07-13

**Authors:** Matthias Weber, Sibylle Banaschak, Markus Alexander Rothschild

**Affiliations:** 1 Institute of Legal Medicine, Faculty of Medicine, University of Cologne, Melatengürtel 60/62, 50823 Cologne, Germany; 2 Landeskriminalamt Nordrhein-Westfalen, Forensic Institute, Völklinger Str. 49, 40219 Düsseldorf, Germany

**Keywords:** Sharp force, Tool marks, Katana sword, Comparison microscope

## Abstract

This paper describes the variety of information that a tool mark analysis on human tissue can provide based on a case of multiple sharp violence. The perpetrator attacked the victim with a sharp-edged weapon against the head, leaving several deep wounds on the back of the skull bone. Three of those marks on the skull bone could be used for a forensic tool mark examination. Silicone casts of the marks were compared by light microscopy with casts of test marks of Japanese katana swords found at the crime scene. One of the swords could be identified as the one responsible for the marks. In addition, the marks and the test marks were scanned in 3D and examined in a visual on-screen comparison confirming the results from the light microscopic examination. Furthermore, a mathematical approach in which the signatures of the marks from the skull bone and the test marks from the sword were compared by cross correlation confirms those findings. In addition, the aforementioned results were used to determine the orientation of the sword in relation to the cranial bone at the time of the respective impact.

## Introduction

A majority of homicides and manslaughter cases are committed with sharp force. The most commonly used type of weapon is a single-edged, flat-bladed kitchen, pocket or folding knife [1]. Other weapons, tools, and objects like axes, machetes, screwdrivers, broken bottle necks, and swords can also be found.

In all these cases, the object causing injury to the victim is of critical importance to the investigation. Therefore, if a potential weapon is seized, it is the next step to prove that it was actually used to attack the victim. Usually, DNA analysis together with the medicolegal findings will prove that the seized object is the murder weapon.

If the body of the victim shows tool marks like stab or cut injuries on solid tissue (bone, cartilage), it is also possible to identify the weapon through a microscopic tool mark examination [2–7]. If the tool marks are of sufficient quality, they can be used to prove that the weapon was in direct contact with the victim’s body. In the best case, the tool mark examination can provide additional information valuable for the crime scene reconstruction. DNA analysis, on the other hand, can prove that cell material of the perpetrator was found on the handle of the weapon and blood of the victim on the blade. The results of both examinations provide a conclusive chain of evidence.

In this case report, two Katana swords with blood stains were secured at the crime scene. The microscopic examination of the tool marks on the skull bone made it possible to identify one of the swords as having made the injuries. In addition, it provided information on the angle of attack and the distance between perpetrator and victim when the strikes to the head occurred.

The results of the light microscopic comparison were supported by the computational comparison of the signatures of the marks from the bone and test marks from the sword.

## Case

### Case scenario

The main trial in court revealed that two men met on a bus. After a conversation about their common passion to play chess, they decided to go on a chess tournament together. Both men were heavy drinkers, and on the way to the tournament, they started to consume alcohol. In the end, both were inebriated and not in the condition to play chess. They went to the offender’s house to continue drinking. There, the two men must have had an argument during which the offender attacked the victim. Only a few details about the crime itself are known, but in the end, the victim suffered a number of deep blow wounds and died. After the course of the crime, the police were called in by the perpetrator himself.

### Crime scene and blood stain pattern

The crime scene was in a small town in the semidetached house of the perpetrator. When the police entered the crime scene, they found the victim in the living room. His body lay on the floor in front of a couch on its side with arms and legs bent. The back of the head pointed upwards and showed numerous deep injuries.

At the scene, the forensic pathologist together with the police investigators observed a pool of blood next to the body, most of it around the head area. There was also a large amount of blood on the couch. Besides this passive blood spatter, impact stains resulting from blood projecting through the air were found. The highest amount of projected spatter was found on the floor, the couch, and the wall near the head of the victim. There were no blood stains on the ceiling but on several objects near the body in different directions. Further signs of a dynamic event were not visible. All the observations support the hypothesis that the victim fell over and has been further attacked in this position.

**Fig. 1:**
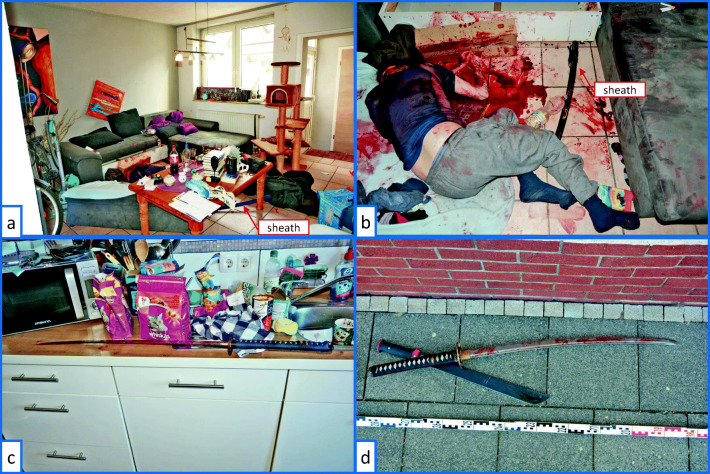
**a** Overview of the living room; the victim was found lying between the couch and a coffee table; a blue katana sheath is located underneath the coffee table. **b** Position in which the victim was found; a black katana sheath is located between the body and the couch. **c** Location of a katana sword with a black handle and a machete on the sidewalk. **d** A katana sword with a blue handle is found in the kitchen on the sink

Right next to the victim, the sheaths of two Katana swords were found (Fig. 1a, b). During the search of the house, a bloodstained sword was found in the kitchen (Fig. 1c). Another bloodstained sword and a machete were found on the sidewalk just outside the house (Fig. 1d).

## Autopsy examination

The autopsy revealed a minimum of 23 sharp force wounds on the back of the head and neck: five parallel incisions with various defects (“chipping”) to the skull, one longitudinal incision and almost complete cut on the right ear, one longitudinal incision on the left side of the occipital bone, and 16 partially intersecting incisions with bone grafts on the occipital bone without opening the skull. However, no intracranial nor intracerebral hemorrhages were detected. The muscles of the back of the neck (mm. trapezius; mm. splenius capitis; mm. semispinalis capitis) were completely severed by at least two cuts, accompanied by two bone grafts of the skull base. The dura mater and cervical spine were intact. Three more incisions of the skin of the neck, three incisions on the right shoulder (partly on the back), and three partly deeper incisions on the right arm were found. The victim showed typical defensive injuries on both hands (more intense on the right hand than on the left hand). Minimal livores and anemia of the internal organs were found as signs of relevant blood loss. Signs of blunt force were also noted such as hematomas of the upper and lower lip, hematomas on the inside of the upper arm, a shaped hematoma under the left nipple, and a subcutaneous hematoma at the base and around the left shoulder blade. No pathological changes of the internal organs were found. There was an intense smell of alcohol of the inner organs. The toxicological examination of the victim revealed a blood alcohol concentration (BAC) of 0.378%.

## Tool mark examination

### Method of tool mark examination

In general, the main goal of a tool mark examination is to determine if a mark was produced by a particular tool. The identification of a tool is possible when the surface of the tool has microscopic irregularities due to the fabrication process (e.g., grinding features) augmented by subsequent wear. These microscopic burrs, dents, and damages can be considered individual specific for one particular tool. The cutting edge of a blade produces a tool mark consisting of parallel edges and grooves produced by those irregularities. If the cutting mark shows a sufficient amount of detail, it is possible to identify that particular blade is the one that has produced the mark [8].

### Swords

**Fig. 2:**
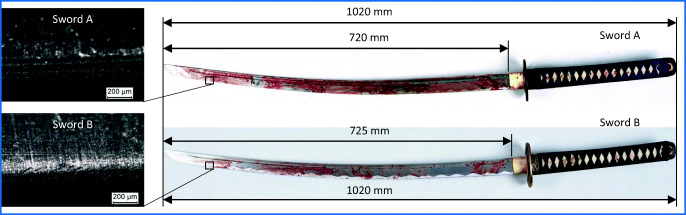
Katana swords A and B (right), grinding structure of the cutting edges of the blades (left)

Two bloodstained swords, known as Japanese katana^1^ swords, were secured by the police. The swords were designated A and B due to their similarities in design and dimensions (Fig. 2). The sword found in the kitchen of the perpetrator’s apartment was designated A. The one secured on the sidewalk near the apartment was designated B. The machete, which was also found on the sidewalk, was not bloodstained and therefore not examined.

Sword A has an overall length of 1020 mm. The blade is 720 mm long with a maximum thickness of 7.0 mm and a maximum width of 31 mm. The handle is wrapped with blue fabric and has an oval guard. Sword B has an overall length of 1020 mm. The blade is 725 mm long with a maximum thickness of 7.5 mm and a maximum width of 32 mm. The handle is wrapped with black fabric and has an oval guard.

The cutting edges of the blades of sword A and B are both relatively sharp and show a grinding perpendicular to the main axis. Blade B shows marks that cover parts of the original grinding structure, running parallel to the cutting edge. Those features could have been produced by manual sharpening. The cutting edges of both blades have wear- and use-related defects, e.g., scratches and dents. Overall, both swords show, due to manufacturing processes and use, a sufficient number of individualizing features on the blades and are therefore suitable for a comparative tool mark examination.

### Cranial bone

**Fig. 3:**
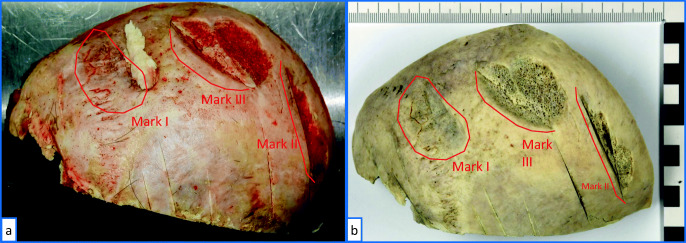
Section of the calvaria with several tool marks. The relevant marks I, II, and III are marked: **a** after autopsy and **b** after maceration process

During autopsy, a section of the calvaria of the cranial bone was removed, rinsed with cold water, blotted dry, and microscopically examined. The sample showed a total of fifteen tool marks, including eight relatively shallow marks and seven marks that cut through the cortical bone into the diploë. All marks were microscopically pre-checked for details suitable for a comparative tool mark examination. Three of the deeper marks, designated I, II, and III (Fig. 3), consisted of straight and parallel running, sharply detailed grooves and edges and showed a sufficient quality for a tool mark examination.

Mark I runs at a shallow angle through the cortical bone over an area of approx. 33 mm × 45 mm and was probably created by a tangential blow that sharply chipped off parts of the cortical bone. Mark II runs approximately perpendicular to the surface through the cortical bone and ends in a depth of approximately 12 mm in the trabecular bone. Mark III runs similar to mark II, in a shallower angle of approximately 45°. Marks II and III show arc-shaped areas of straight, parallel grooves and edges. All three marks have clearly been produced by blows with sharp-edged blades and show a sufficient number of features to be suitable for a tool mark examination.

### Casting and test marks

Marks I, II, and III were recovered with AccuTrans® casting material, which is a low-viscosity, thixotropic elastic casting material, widely used in the field of tool marks, and has been successfully used before by the authors for casting tool marks on tissue [7]. Subsequently, the section of the cranial bone was macerated in a solution of water and washing powder at a maximum temperature of 75 °C until all soft tissue could be carefully brushed off. The sample was degreased using acetone and dried completely before marks I, II, and III were recovered by AccuTrans® casting material once more.

**Fig. 4:**
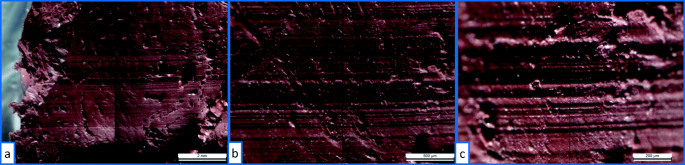
Microscopic comparison of the casts before (left side) and after maceration (right side) as **a** overview and **b** and **c** at high magnification, even the finer striations are not altered by the maceration process

The casts before and after maceration were compared with a Leica FS-C comparison light microscope.^2^ The comparative examination of the casts reveals that the maceration had no considerable effect on the quality of the tool marks in the bony tissue (Fig. 4).

In the next step of the tool mark examination, the bloodstained blades of the katana swords were rinsed with cold water and dried with paper tissue. Test marks of both swords A and B were made in dental wax sheets (Cavex Set Up Wax, hard). To cover every potentially possible cut mark, the blades of the swords were partitioned into four sections, and for each section, two marks (left and right side of the blade) were produced by pushing the cutting edge of the section in the wax sheet. For sword blows, the angle of attack does not have to be varied when making test marks. A blow is performed by swinging the blade. The direction of movement of the blade is therefore orthogonal to the main axis and could only run in another angle if surface and tool were moved significantly relative to each other. A variation of the side angle is also not necessary, since a sword—due to its geometry—only cuts parallel to the surface of the blade.

All test marks were recovered by AccuTrans® casting material, and the information about sword (A/B), section (1 to 4), side (left/right), and orientation of the sword (tip/handle, cutting edge/back of blade) was noted on each cast.

### Light microscopic comparison of cast marks

The casts of marks I, II, and III (victim) and the test marks of the swords were examined using a forensic comparison light microscope (Leica FS-C) with oblique lightning.

**Fig. 5:**
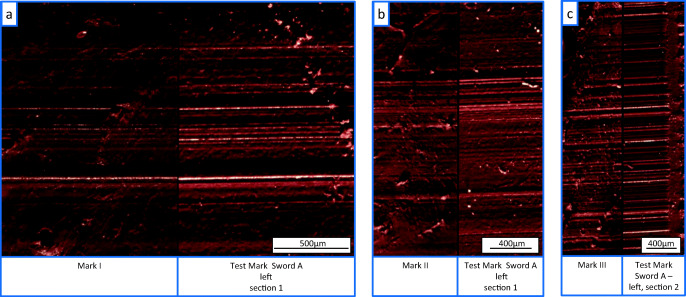
Light microscopic comparison of the casts of **a** tool mark I, **b** tool mark II, and **c** tool mark III compared with the test marks made with sword A

All three marks showed straight and parallel striae (grooves and edges) a very good level of matching with the features of the test marks of sword A (Fig. 5). According to the ENFSI conclusion scale,^3^ this degree of agreement allows the conclusion that sword A is the source of these marks.

When counting the consecutive matching striae (CMS) according to the method of Biasotti [9], groups of more than eight CMS were found by comparing the casts of the marks I, II, and III and the test marks of sword A which also lead to an identification of sword A.

### Optical comparison of 3D scanned marks

**Fig. 6:**
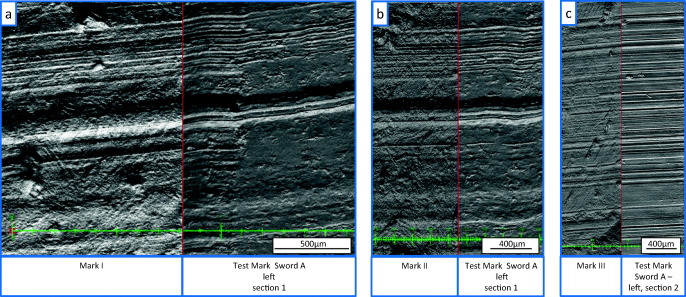
Software based on screen comparison of the topographic data of **a** tool mark I, **b** tool mark II, and **c** tool mark III compared with the test marks made with sword A

In addition to the light microscopic comparison, the casts of the marks and test marks were scanned using a ToolScan (LIM Laboratory Imaging) forensic 3D scanning device. The ToolScan device combines coarse surface features, determined with a laser scan of the surface, with fine features, calculated from a set of photographs of the circumferential illuminated surface (3 μm/px). The scans of marks I, II, and III and test marks were examined on screen using the 3D comparison software LIM Lucia Forensic. The 3D comparison leads to the same conclusion as the light microscopic comparison. The striations of marks I, II, and III matched in a sufficient way with their reference test marks of sword A (Fig. 6). Based on these findings, sword A was identified as the one that produced the marks.

### Computational comparison of 3D scanned marks

Another approach for the comparison of marks and test marks is the application of 3D methodologies to acquire characteristic information about striated marks [10–15]. After the scanning process, a signature is generated which corresponds to the cross section of the striated tool mark. In the pre-processing, all unreliable data points, e.g., outliers, are identified. Bachrach et al. [11] eliminated inaccurately measured data points by two approaches. The first approach was to compare the slope between a data point and its neighbor with a pre-established threshold. The second approach was to identify outliers as data points deviating a pre-determined number of standard deviations with respect to the local mean. Baiker et al. [12, 13] substituted invalid measurement points and local peaks using 10 nearest neighbor interpolation. Additionally, they generated the signature of the tool mark by averaging the entire data set along the striations.

The next step is the normalization of the signature, where variations in the topography, as might be expected when the surface of the specimen is not perfectly level to the reference plane of the scanning device, are compensated and the signature is aligned to the x-axis. Bachrach et al. [11] used a Gaussian band pass filter, and Baiker et al. [12, 13] used Chebyshev type 2 bandpass filters for normalization and noise reduction. Hadler et al. [15] applied a coarse smooth to the data set to get a normalized version of the tool mark signature.

The last step is the similarity measure of the signatures of the mark and test mark. Bachrach et al. [11] used normalized relative distance metric and reached a mean of 0.33 (STD 0.07) for nonmatching and a mean of 0.92 (STD 0.07) for matching marks. Baiker et al. determined the cross correlation and reached a mean of 0.22 (STD 0.13) for nonmatching and means up to 0.99 (STD 0.01) for matching marks [13].

Although this approach is a promising addition to the traditional light microscopic comparison, since it compares two marks without the subjective influence of the examiner, no work has been published presenting a case study in which the signatures of tool marks on human tissue and test marks have been compared using cross correlation.

**Fig. 7:**
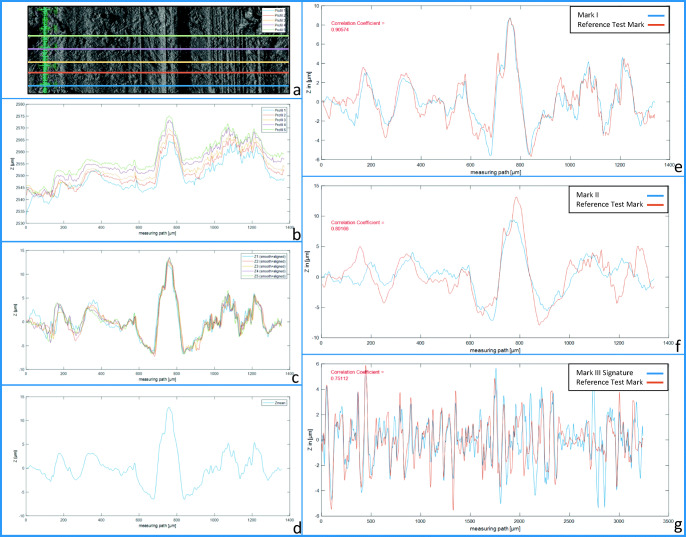
Comparison of striated tool marks by cross correlation. **a** Signature extraction. **b** Five signatures before pre-processing. **c** Five signatures after moving average subtraction and low-pass filter. **d** Five signatures averaged to one. **e** to **g** Signatures of tool mark I, II, and III and signatures of test marks of sword A after alignment by cross correlation

In this case, we used the surface data from the ToolScan device. At first, the data sets of mark and test mark were rotated using the ToolScan software LIM Lucia Forensic 2019 to align the striations. Next, five signatures per mark and test mark were extracted from the data sets in areas of least disturbances and imported into MATLAB (R2018a). In the pre-processing, all signatures were aligned to the x-axis by subtracting the moving average, discarding the low-frequency components and keeping the high-frequency components. Noise reduction was performed by a low-pass filter. In the next step, the five signatures of each mark and test mark were aligned by normalized^4^ cross correlation and then averaged. The averaged signatures of mark and test mark were then compared using normalized cross correlation (Eq. 1: Cross Correlation, Fig. 7a–d ).

1 $$ x\mathrm{corr}\left\{k\right\}=\frac{\sum_{i=1}^N{S}_1\left[i\right]\cdotp {S}_2\left[i+k\right]}{\sqrt{\sum_{i=1}^N{\left({S}_1\left[i\right]\right)}^2\cdotp {\sum}_{i=1}^N{\left({S}_2\left[i+k\right]\right)}^2}} $$

Mark I and the reference test mark of sword A (left side of the blade, section 1) reached a correlation coefficient of 0.91, mark II and the reference test mark of sword A (left side of the blade, section 1) reached a correlation coefficient of 0.80, and mark III and the reference test mark of sword A (left side of the blade, section 2) reached a correlation coefficient of 0.75 (Fig. 7e–g).

**Fig. 8:**
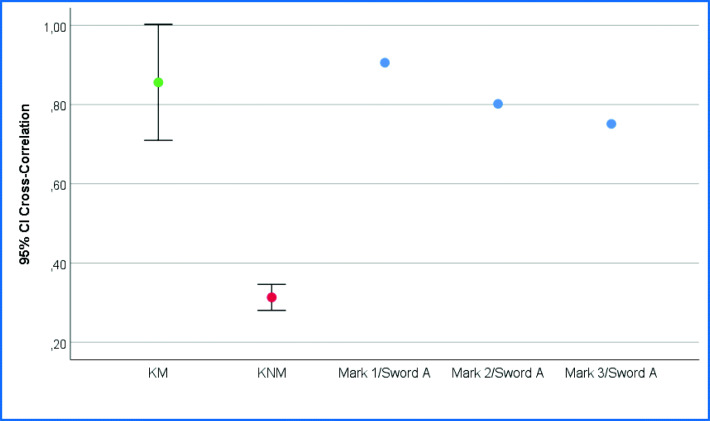
Cross correlation of the signatures of marks I, II, and III and the test marks of sword A and cross correlation of the test knife marks (KM = known matches, KNM = known non-matches)

To classify the results, two cut marks with four identical test knife blades were made manually in wax sheets (Cavex Set Up Modelling wax, regular) and then cast with AccuTrans AB brown (Colthène Whaledent) casting material. The casts of the test marks were scanned with the ToolScan device, and signatures were generated similar to the method described before. The signatures of the two marks of the same knife (known matches = KM, *N* = 4) and the marks of different knives (known non-matches = KNM, *N* = 24) were compared by cross correlation. The results of all KM and all KNM were averaged and shown together with the results of the comparative examination of the marks on the skull bone in Fig. 8. The cross correlation of all three marks (I to III) lies within the 95% confidence interval of the known matches.

### 3D analysis of the tool mark examination results

**Fig. 9:**
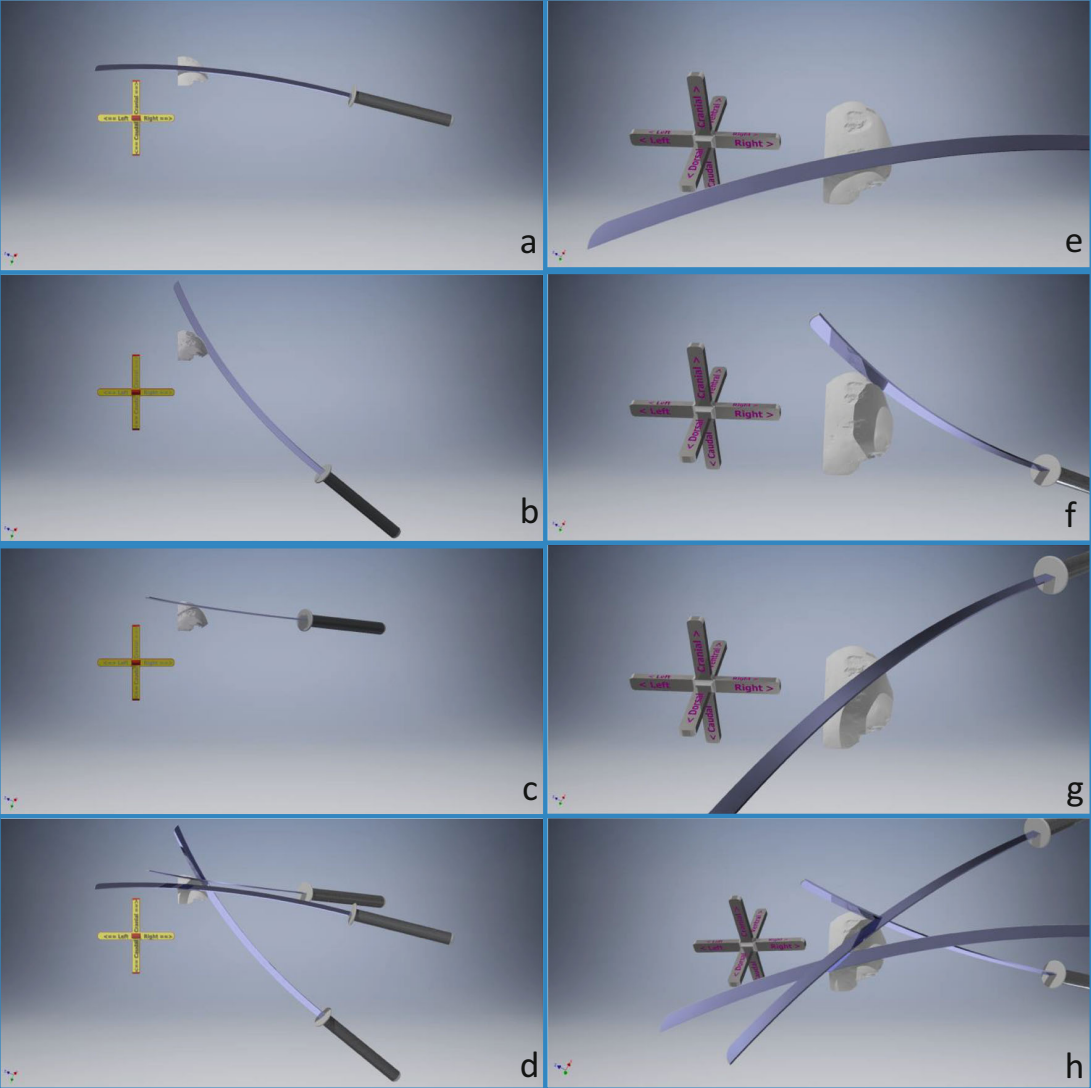
3D-CT model of the cranial bone section and CAD model of sword A. The sword is placed according to the results of the tool mark analysis. Panels a to d (posterior view) show the orientation of the sword when creating mark I (**a**), II (**b**), and III (**c**) and all together in **d**. Panels e to h show close-ups and from a different angle

Since sword A was identified as having produced all three marks on the skull bone, the exact positions on the edge of the sword of the respective areas that have produced the marks are known. Mark I was made with the left side of the blade at a distance of approximately 56 cm from the hand guard of the sword. Mark II was made with the left side of the blade, approximately 54 cm from the hand guard of the sword. Mark III was made with the left side of the blade, approximately 44.5 cm from the hand guard of the sword. Additionally, the relevant side of the cutting edge and the orientation of the sword in relation to the skull bone could be determined. By analyzing the marks on the bone, the direction of movement of each sword blow could be derived. For a better graphical representation of this information, a 3D CT scan of the cranial bone section was created and imported into the CAD software Autodesk Inventor (Professional 2017). A 3D CAD model of sword A was created and oriented to the bone according to the results of the tool mark examination (Fig. 9).

## Discussion

Casts made before and after the maceration process were compared microscopically, and no significant differences were found on the topography of the marks. The extent to which the maceration method and other factors like the fineness of the striations or the properties of the bone material could influence the quality of the marks was not considered in this paper. Those questions should be addressed in future work.

As a result of the light microscopic examination, sword A was clearly identified as having produced marks I, II, and III. Using the commonly referenced Six-Level Conclusion Scale of the ENFSI Expert Working Group Marks [16], the result would be a level one (identification) for this tool mark examination. The optical examination of the 3D scanned marks yielded the same results. All results were checked by another expert. In comparison with light microscopy, the software-supported examination of 3D data has the advantage that the data can be examined simultaneously by several experts at different locations. Further advantages of this examination compared with the light microscopic approach are the software-based features, e.g., settings of the virtual illumination (elevation and azimuth angle, intensity), side-by-side and overlay comparison, and free rotating of the virtual marks. According to the authors’ experience, the classical microscopic comparison is often faster.

From the fifteen tool marks on the skull bone, only three marks were detailed enough for a tool mark examination. One reason for this is that a certain thickness of the bone layer is needed to create an evaluable mark. For the twelve remaining marks, only a comparison of the class characteristics could be carried out. These marks could have been created by either of the two swords or by any object with similar class characteristics.

As an additional approach, the marks were computationally compared by the cross correlation of their signatures. The correlations obtained were compared with known matches and known non-matches and are clearly in the range of the known-matches, supporting the identification of sword A. This mathematical approach has the advantage of providing a more objective measure and comparison of the feature than the optical microscopic comparison. It should be noted that the approach compares signatures without distinguishing between class, subclass, and individual characteristics. For signatures of tools with distinctive pronounced class or subclass characteristics compared with their individual characteristics, e.g., serrated knives, the approach could yield high cross correlation even for non-matches. Another disadvantage of this method is that disturbances or differences in the marks, which would be ignored by an expert’s visual perception, interfere with the mathematical comparison. Disturbances can be compensated by averaging a larger number of signatures per mark. However, in real case work, the tool marks are often only a few mm long, so it is not possible to generate a high number of signatures. This means that the signature used for comparison is only averaged from a few individual signatures and therefore disturbances have a comparatively greater influence.

Differences between marks and test marks, resulting from changes of the tool due to use, cannot be compensated for and could lead to false negative results in the computational comparison. Another critical aspect of this approach is that the setting of the filters can have a great influence on the cross correlation. If, for example, the low frequencies of two marks that are unequally oriented to the reference plane of the scanning device are not sufficiently filtered, the cross correlation yields very low results even for non-matches. This topic can be examined in future work to define standards for the filter settings of tool mark examinations. Nevertheless, the approach is an excellent complement to the traditional light microscopic approach.

As a result of the optical analysis of the marks on the skull bone and the comparative tool mark examination, it was possible to determine the side and exact locations on the cutting edge of the sword that produced the marks on the bone. These results gave valuable information of the relative positions of attacker and victim at the time of the attacks which led to the tool marks. Given the orientation of the marks, the location, and side on the cutting edge, it can in this case be stated that the blows that lead to mark I, II, and III were executed from the right side of the victim. Those results are in good agreement with the results of the autoptic examination and the findings at the crime scene.

## Conclusion

In this article, we present the comparative tool mark examination in a murder case involving sharp force trauma to the cranial bone. Two potential murder weapons, Japanese katana swords, were secured at the scene of the crime and in the vicinity. Which or if both swords caused the injuries to the skull bone was unknown. Three evaluable tool marks were cast on the cranial bone.

Overall, the results of this study confirm that bone material is capable of reproducing highly detailed tool marks of a quality sufficient for identification of the murder weapon. In addition, the results of the tool mark examination can contribute valuable information to the reconstruction of what happened at the scene of the crime.

A report on the results of this investigation was presented during the court proceedings. The perpetrator was sentenced to 9 years in prison for manslaughter and to a court-ordered rehab.

## Footnotes

1 Katana: single-edged sword with a curved blade. The handle is long enough to be used as a two-hand sword and has a squared or circular guard. Katanas were the traditional swords of the Samurai.

2 Two compound light microscopes which allow the user to view two specimens at the same time in a side-by-side view

3 Six-Level Conclusion Scale for Shoeprint and Toolmarks Examinations of the ENFSI (European Network of Forensic Science Institutes) Expert Working Group “Marks”

4 Autocorrelation at zero lag equals 1.
