# Correction to: Sharp force trauma with two katana swords: identifying the murder weapon by comparing tool marks on the skull bone

**DOI:** 10.1007/s00414-021-02657-1

**Published:** 2021-08-18

**Authors:** Matthias Weber, Sibylle Banaschak, Markus Alexander Rothschild

**Affiliations:** 1 Institute of Legal Medicine, Faculty of Medicine, University of Cologne, Melatengürtel 60/62, 50823 Cologne, Germany; 2 Landeskriminalamt Nordrhein-Westfalen, Forensic Institute, Völklinger Str. 49, 40219 Düsseldorf, Germany

**Correction to: International Journal of Legal Medicine (2021) 135:313–322**

10.1007/s00414-020-02372-3

The article “Sharp force trauma with two katana swords: identifying the murder weapon by comparing tool marks on the skull bone”, written by Matthias Weber, Sibylle Banaschak and Markus Alexander Rothschild, was originally published Online First without Open Access. After publication in volume 135, issue 1, page 313–322 the author decided to opt for Open Choice and to make the article an Open Access publication. Therefore, the copyright of the article has been changed to © The Author(s) 2020 and the article is forthwith distributed under the terms of the Creative Commons Attribution 4.0 International License, which permits use, sharing, adaptation, distribution and reproduction in any medium or format, as long as you give appropriate credit to the original author(s) and the source, provide a link to the Creative Commons licence, and indicate if changes were made. The images or other third party material in this article are included in the article’s Creative Commons licence, unless indicated otherwise in a credit line to the material. If material is not included in the article’s Creative Commons licence and your intended use is not permitted by statutory regulation or exceeds the permitted use, you will need to obtain permission directly from the copyright holder. To view a copy of this licence, visit http://creativecommons.org/licenses/by/4.0.

The original article has been corrected.

